# Supplementary material for: Polyketide synthase gene clusters in the ericoid mycorrhizal fungus Oidiodendron maius: genome-wide prediction, expression profiling, and functional characterization of knockout mutants
Source: Front Plant Sci. 2026 Sep 16;17:1924430. doi: 10.3389/fpls.2026.1924430 (PMC13623903; doi:10.3389/fpls.2026.1924430)
Supplement: Supplementary file 1 [file SupplementaryFile1.zip › Supplementary Materials/Supplementary Folder 1.zip!/Oidma1_AssemblyScaffolds_Repeatmasked/knownclusterblast/cluster54/jgi.p_Oidma1_107026_mibig_hits.html]

| MIBiG Protein | Description | MIBiG Cluster | MiBiG Product | % ID | % Coverage | BLAST Score | E-value |
| --- | --- | --- | --- | --- | --- | --- | --- |
| ADE34525.1 | SsfF | BGC0000269 | SF2575\_biosynthetic\_gene\_cluster | 35 | 96.9512195122 | 159 | 6.2e-39 |
| CCA54216.1 | Aldo-keto\_reductase | BGC0000893 | Chloramphenicol\_biosynthetic\_gene\_cluster | 38 | 102.43902439 | 155 | 1.2e-37 |
| EFG07997.1 | Aldo/keto\_reductase | BGC0000843 | Clavams\_biosynthetic\_gene\_cluster | 35 | 100.609756098 | 150 | 2.9e-36 |
| AFH74311.1 | Cvm1-like\_aldo/keto\_reductase | BGC0001151 | Clavams\_biosynthetic\_gene\_cluster | 32 | 102.43902439 | 135 | 1.2e-31 |
